# Automated identification of retinopathy of prematurity by image-based deep learning

**DOI:** 10.1186/s40662-020-00206-2

**Published:** 2020-08-01

**Authors:** Yan Tong, Wei Lu, Qin-qin Deng, Changzheng Chen, Yin Shen

**Affiliations:** 1 Eye Center, Renmin Hospital of Wuhan University, Wuhan, 430060 Hubei China; 2 Medical Research Institute, Wuhan University, Wuhan, Hubei China

**Keywords:** Deep learning, Retinopathy of prematurity, Artificial intelligence, Fundus image

## Abstract

**Background:**

Retinopathy of prematurity (ROP) is a leading cause of childhood blindness worldwide but can be a treatable retinal disease with appropriate and timely diagnosis. This study was performed to develop a robust intelligent system based on deep learning to automatically classify the severity of ROP from fundus images and detect the stage of ROP and presence of plus disease to enable automated diagnosis and further treatment.

**Methods:**

A total of 36,231 fundus images were labeled by 13 licensed retinal experts. A 101-layer convolutional neural network (ResNet) and a faster region-based convolutional neural network (Faster-RCNN) were trained for image classification and identification. We applied a 10-fold cross-validation method to train and optimize our algorithms. The accuracy, sensitivity, and specificity were assessed in a four-degree classification task to evaluate the performance of the intelligent system. The performance of the system was compared with results obtained by two retinal experts. Moreover, the system was designed to detect the stage of ROP and presence of plus disease as well as to highlight lesion regions based on an object detection network using Faster-RCNN.

**Results:**

The system achieved an accuracy of 0.903 for the ROP severity classification. Specifically, the accuracies in discriminating normal, mild, semi-urgent, and urgent were 0.883, 0.900, 0.957, and 0.870, respectively; the corresponding accuracies of the two experts were 0.902 and 0.898. Furthermore, our model achieved an accuracy of 0.957 for detecting the stage of ROP and 0.896 for detecting plus disease; the accuracies in discriminating stage I to stage V were 0.876, 0.942, 0.968, 0.998 and 0.999, respectively.

**Conclusions:**

Our system was able to detect ROP and differentiate four-level classification fundus images with high accuracy and specificity. The performance of the system was comparable to or better than that of human experts, demonstrating that this system could be used to support clinical decisions.

## Background

Retinopathy of prematurity (ROP) is a proliferative retinal vascular disease that affects approximately two-thirds of premature infants who weigh less than 1250 g at birth. It is associated with abnormal retinal vascular development at the boundary of vascularized and avascular peripheral retina [1, 2]. Worldwide, an estimated 30,000 premature infants annually experience blindness or severe loss of vision due to ROP [3]. Most cases of ROP are mild and resolve spontaneously without intervention; 5 to 10% of cases progress to more severe ROP, which can lead to retinal detachment or distortion of the retina and permanent blindness if left untreated [4]. Whereas clinical diagnosis and early disease detection remain subjective; high levels of inconsistency in ROP diagnosis have been observed even among ROP experts [5, 6]. Therefore, it is urgent to establish a screening tool that can rapidly identify fundus images requiring further attention and critical analysis by ophthalmologists, thereby increasing the accuracy and efficiency of diagnosis.

Artificial intelligence (AI), inspired by the multilayered human neuronal system, has achieved great performance within medical imaging interpretation and triage tasks, allowing clinical experts to diagnose diseases efficiently and untrained technicians to objectively screen more patients. Deep learning (DL) has significantly extended the capabilities of images classification, object detection, drug discovery, and robot functions [7]. Convolutional neural networks (CNNs) are DL algorithms commonly applied in image classification, which have been successfully used in the diagnosis of skin cancer [8], lung cancer [9], glioma [10], and breast histopathology [11]. DL has achieved automated detection of retinal diseases [12, 13], including diabetic retinopathy [14], glaucoma [15], age-related macular degeneration, and cataracts [16]. Recently, several studies regarding the diagnosis of ROP with AI have achieved promising results. Approaches for automated identification of plus disease in ROP depend on traditional approaches, such as machine learning with handcrafted features [17]. Gelman established a computer-based image analysis system to distinguish plus disease with 95% accuracy, which is comparable with expert diagnosis [18]. Brown et al. developed an algorithm based on DL to automatically distinguish the presence of plus disease or pre-plus disease with high sensitivity and specificity [19]. However, the above-mentioned studies were mainly focused on plus disease in ROP. An automated ROP diagnosis system that can analyze real-world clinical features (i.e., stage and zone of ROP as well as the presence of plus disease) is rare.

In this study, we established an intelligent system to achieve detection and classification of ROP in fundus images. The purpose of our study was to: (1) implement and evaluate a CNN-based DL system for four-level diagnosis (normal, mild, semi-urgent, urgent) of ROP in fundus images; (2) determine the accuracy of the system by comparing its diagnostic performance with that of experienced retinal experts; (3) detect the stage of ROP and presence of plus disease, and predict lesion location in fundus images using a faster region-based convolutional neural network (Faster-RCNN).

## Methods

### Ethics approval

Collection and labeling of fundus images were performed by ophthalmologists at Renmin Hospital of Wuhan University Eye Center. This study followed the tenets of the Declaration of Helsinki [19], and was approved by the institutional review board of Renmin Hospital of Wuhan University (ID: WDRY2019-K032). For all involved patients, written informed consent was obtained from their parents for imaging and study participation. In addition, we deleted all patients’ sensitive information prior to image viewing, to ensure that their personal information remained anonymous and confidential.

### Data sets

For algorithm development, a total of 38,895 fundus images from the ROP screening (from February 1, 2012, to October 1, 2016) were retrospectively collected from Renmin Hospital of Wuhan University Eye Center. All images were obtained using a wide-angle imaging device (RetCam; Clarity Medical Systems, Pleasanton, CA). The resolution of the image is 640 × 480 pixels. The dataset also included follow-up images from the same patients who underwent ROP screening.

### Image labeling, preprocessing and dataset division

**Fig. 1:**
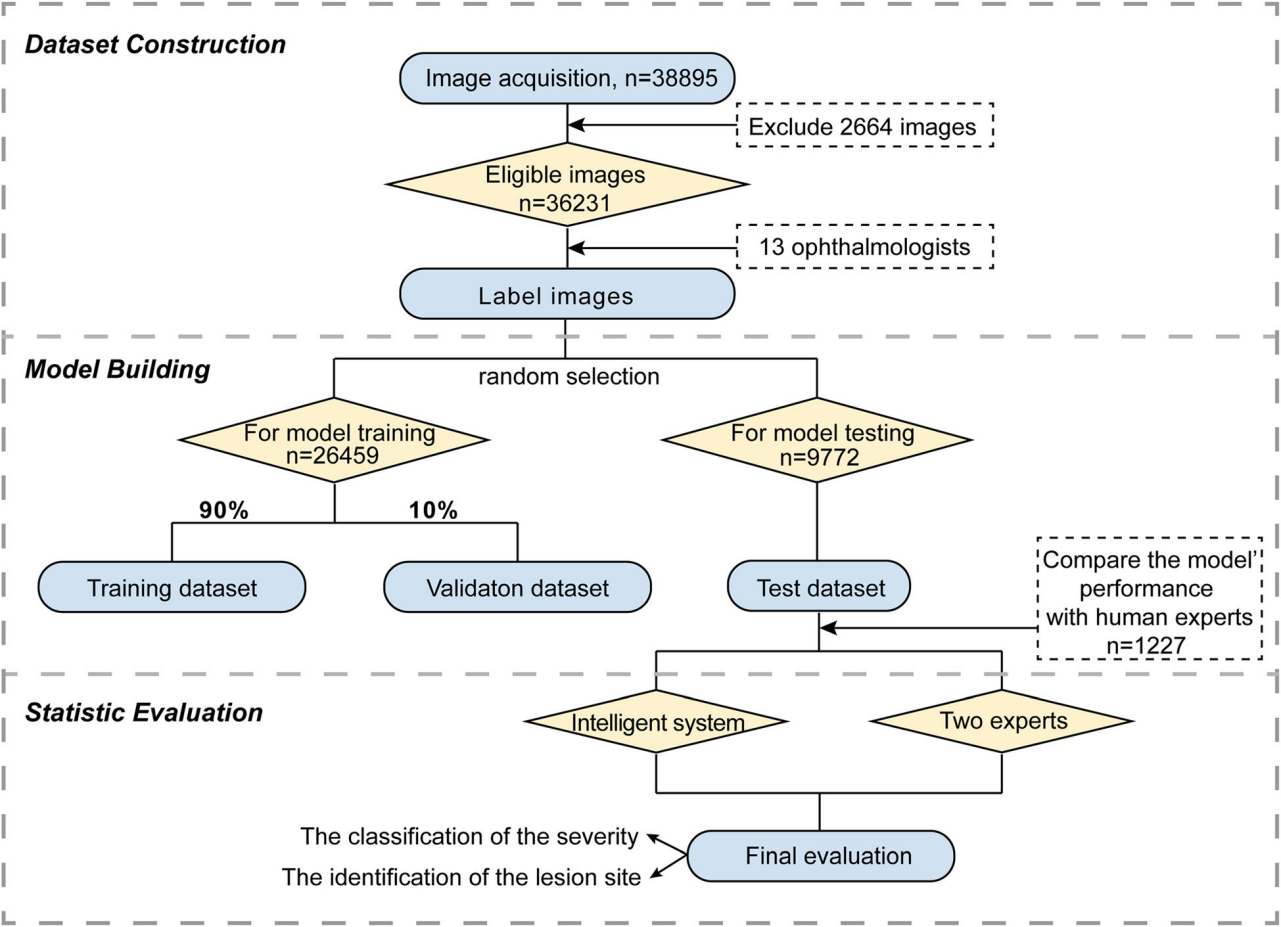
Workflow of image labeling and model training

The overall experimental design and dataset selection process is shown in Fig. 1. The current study invited 13 licensed ophthalmologists, who specialized in retinal diseases diagnosis. Images were randomly assigned to 11 junior retinal experts for first-round screening and labeling. In the second round, the remaining two senior retinal experts who have over 10 years of individual clinical experience were invited to confirm (or correct) the labeling results. A total of 2664 images were excluded based on the following exclusion criteria: (1) poor image quality; (2) imaging artefacts; (3) unfocused scans; (4) presence of other disease phenotypes (e.g., retinal hemorrhage). No images were excluded based on age, sex, or race. Eventually, the remaining 36,231 images were included in the current study to build the intelligent system.

**Fig. 2:**
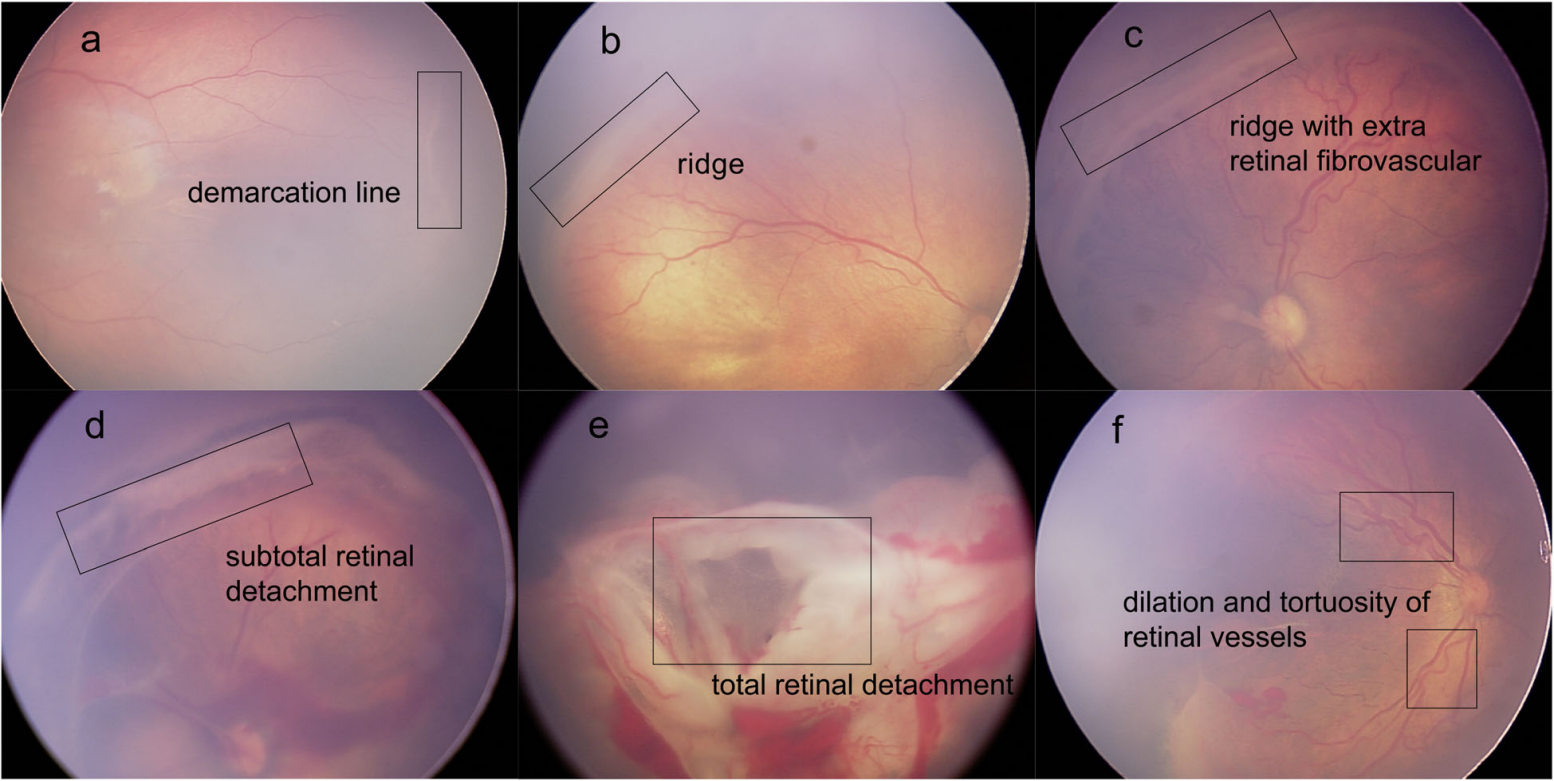
Representative ROP images with annotations of five stages and the presence of plus disease. Box outlines in (**a**-**f**) indicate lesion sites. (**a**) Stage I: Demarcation line. (**b**) Stage II: Ridge. (**c**) Stage III: Ridge with extra retinal fibrovascular involvement. (**d**) Stage IV: Subtotal retinal detachment. (**e**) Stage V: Total retinal detachment. (**f**) Plus disease: Dilatation and tortuosity of retinal vessels

Each image was annotated with two labels: the classification label and the identification label. The classification labels constituted one of four degrees of ROP severity according to the requirements of clinical treatment [20]: “normal” (no abnormalities); “mild” (stage I or stage II, without plus disease; routine observation); “semi-urgent” (stage I or stage II, with plus disease; suggested referral); and “urgent” (stage III, stage IV, or stage V, with or without plus disease; urgent referral for treatment). The identification labels were added to indicate ROP stages: “demarcation line,” “ridge,” “ridge with extra retinal fibrovascular involvement,” “subtotal retinal detachment,” and “total retinal detachment”; the identification labels were also added to indicate plus disease: “dilation and tortuosity of retinal vessels”, based on the International Classification of Retinopathy of Prematurity system [21]. In addition, the lesion area was delineated with a box outline by the retinal experts. Representative ROP images are shown in Fig. 2. In addition, the experts labeled optic disc, fovea, and laser scar on the images to assist in diagnosis and monitoring of any therapeutic effects.

To account for image variation within our dataset, we used preprocessed versions of the original images and normalized the image before learning. The preprocessing steps consisted dataset augmentation followed by resizing. Data augmentation was a method that used image transformations across a sample dataset to increase image heterogeneity while preserving prognostic characteristics of the image itself. Since the fundus diagnosis primarily depended on the identification of major anatomical structures, regardless of orientation, we encoded rotational invariance into our predictions by randomly rotating images before propagating these images into our model. To preprocess images further before learning, we augmented the dataset by adding random noise and adjusted the image brightness. The large dataset improves the generalization of the model and reduces overfitting. Images were then downsized to a standard resolution of 224 × 224 pixels to fit the expected input size for algorithm training.

We randomly divided the obtained processed dataset into the training and test datasets. The training dataset was used to develop the learning model, while the test dataset was used to evaluate the model. Image numbers of each category in the training and test datasets are summarized in Table 1. During the training process, we used a conventional 10-fold cross-validation [22] method to evaluate and optimize our model. The sample was randomly partitioned into 10 complementary subsamples of equal size. Nine folds were selected as the training set and one was selected as the validation set over 10 iterations. Therefore, 90% of the data was used for training and 10% of the data was used for validation. In this context, all patients in the dataset participated in a validation, and each was predicted exactly once before the algorithms were ready to be tested.

**Table 1:** Number of images in the training and test datasets

Clinical features	Definition	Training dataset	Test dataset
Stage I	Demarcation line	1687	377
Stage II	Ridge	263	77
Stage III	Ridge with extra retinal fibrovascular involvement	179	48
Stage IV	Subtotal retinal detachment	45	13
Stage V	Total retinal detachment	10	4
Plus disease	Dilation and tortuosity of retinal vessels	2745	261
Optic disc	–	12,383	5278
Fovea	–	8455	3568
Laser scar	–	692	146
Total	–	26,459	9772

### Development of the algorithm

In this study, we used two deep CNNs: the 101-layer ResNet (classification network) and the Faster R-CNN (identification network). The model was built and trained with the Keras package in Python programming language (ver. 2.7.9, Python Software Foundation, Beaverton, US) using the TensorFlow backend (http://www.tensorflow.org). To improve the training speed, we utilized a ResNet-101 CNN architecture that was pre-trained on the ImageNet (http://www.image-net.org) database of 1.4 million images [23], and retrain it on our dataset using transfer learning, by which an algorithm can apply cumulative knowledge learned from other datasets to a new task [24]. The CNN consisted of multiple convolutional layers that learned local features of images and generated classifications. It included pooling layers (average pool and max pool) that merged semantically similar features into one feature, thereby reducing the dimensionality of the extracted features and fully connected layers to combine these features and provide a final probability value for the class. The original code of the study is available at https://github.com/whu-eyelab/Rop_.

**Fig. 3:**
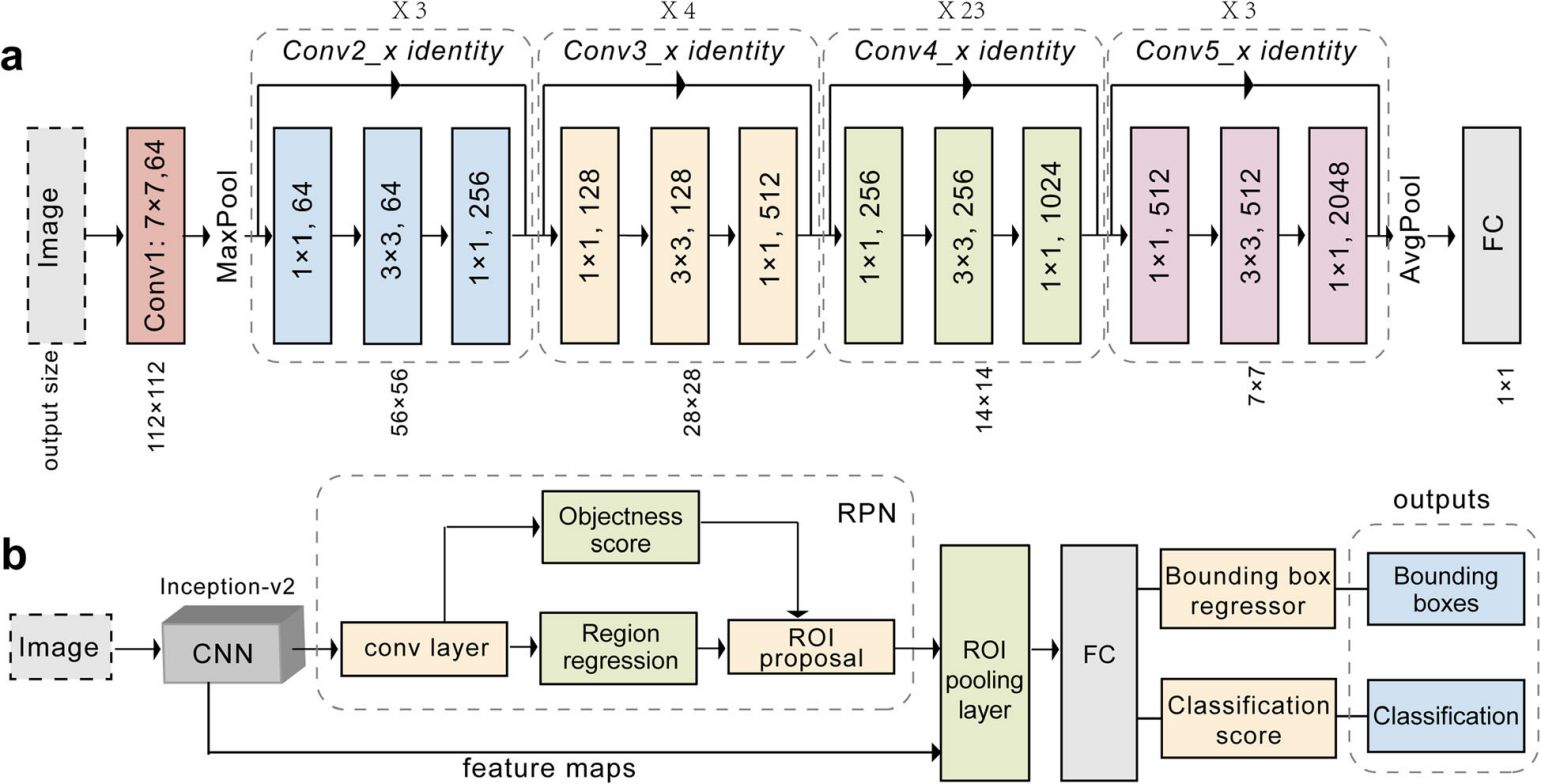
Workflow diagram. **a** Typical architecture of the 101-layer ResNet. **b** The flowchart of Faster-RCNN. Abbreviations: Conv, convolutional layer; RPN, region proposal network; ROI, region of interest; AvgPool, average pool; FC, full connected layer; CNN, convolutional neural network; Faster-RCNN, faster region-based convolutional neural network

Recent studies have shown that network depth is beneficial to classification accuracy [25]. However, as the network gains greater depth, its performance becomes saturated and then begins to decrease rapidly [26]. The ResNet framework can correct this problem. Throughout the deep network, shortcut connections are added every three convolutional layers. These shortcut connections perform identity mapping without adding extra parameters or increasing the computational complexity, which enhances the ease of optimizing the network during the training process. Therefore, ResNet enables achievement of higher accuracy from deeper networks than from shallower networks when performing image classification tasks. The network was trained with a learning rate of 0.0001 and the computation cost of 7.6 × 10^9^ floating-point operations using the parameters presented in Fig. 3a.

Faster R-CNN is a high-performing object detection model, which was the winning entry of the Common Objects in Context (COCO) detection challenge [27]. Object detection involved recognition and classification of every object in an image as well as positioning each object within a bounding box. We configured Faster R-CNN with a pretrained Inception-ResNet-v2 model provided by TensorFlow Object Detection application programming interface (Fig. 3b) to identify the stage of ROP and the presence of plus disease as well as to predict the objective boundaries of the lesion sites. The pretrained model had been trained on COCO, which was a large image dataset designed for object detection [28]. During the training process, we applied the fine-tuning technique to transfer the connection weights from the pretrained model to our model and retrained the model to the present task. This model accepted an image as input and performed five main assessments: the region proposal network was used to identify object regions in an image; a classifier block of the outline box regressor and an object classifier were used to assess candidate boxes from the output of the region proposal network; region of interest pooling and fully connected layers were the final assessments. Eventually, the model outputs the bounding box of each target object as well as the corresponding category label.

**Fig. 4:**
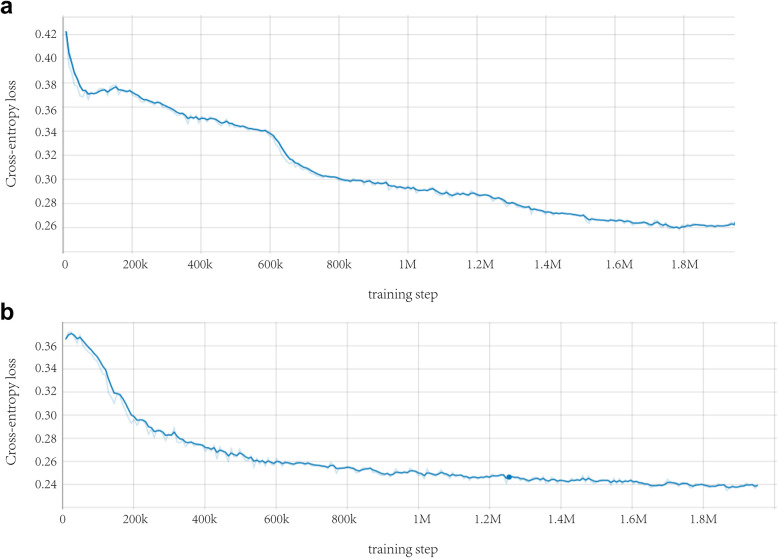
Comparison between the cross-entropy loss function curve and the training step. (**a**) Performance of the training dataset; (**b**) Performance of the validation dataset

**Fig. 5:**
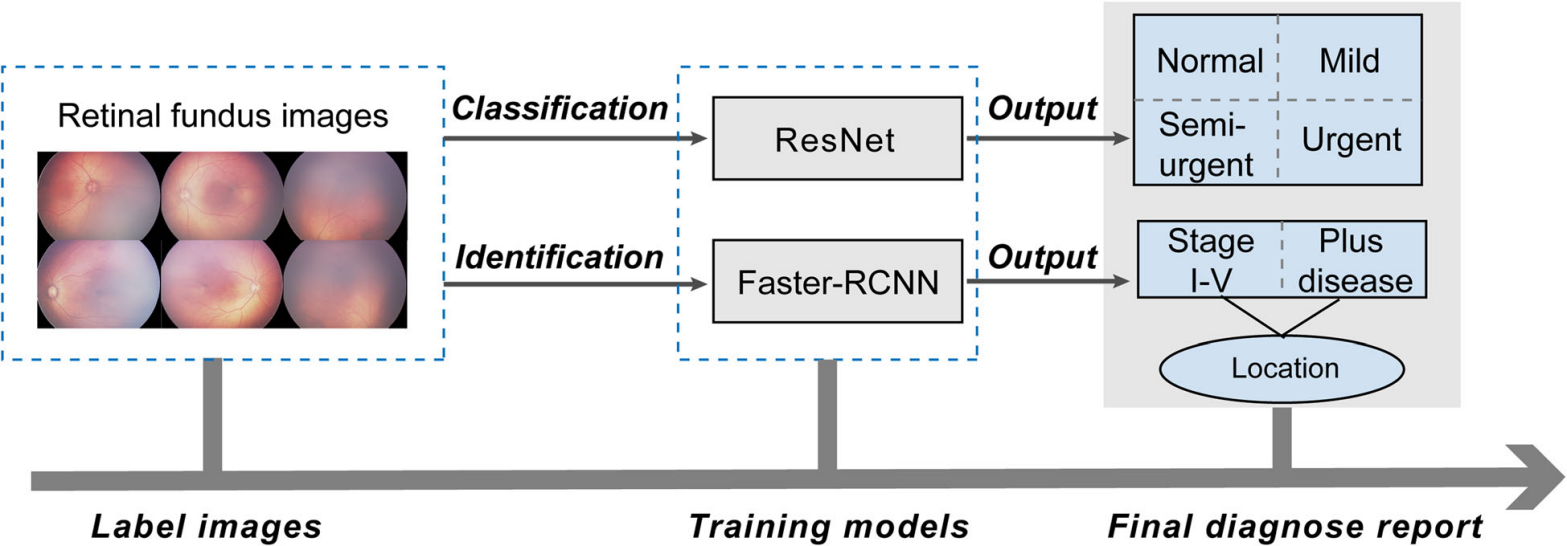
Workflow of our system

We combined these two CNN networks as a system to process a large-scale ROP dataset; the system will eventually output the classification of ROP severity as well as the diagnosis of ROP stage and the presence of plus disease. The intelligent system ran a total of 120 training epochs (iterations) and the training stopped when the cross-entropy loss function was minimized by stochastic gradient descent. Then, the model with the lowest loss (highest accuracy) was selected for use on the test dataset. TensorBoard chart was used to show the performance of model training and validation data (Fig. 4). All classifications produce convergence when training reaches the final layer. Figure 5 shows the overall working system.

### Statistical analysis

To evaluate the performance of the intelligent system, three evaluation indicators were compared, including accuracy, sensitivity, specificity and F1-score. To further evaluate the performance of the system, 1227 fundus images captured during routine clinical ROP screening were used to compare the prediction accuracy of the system within the four-level classification relative to the diagnoses of two experienced human experts on retinal imaging. We also plotted the confusion matrices of the 101-layer ResNet and compared the locations of the lesions predicted by the intelligent system with those labeled by the experts. Statistical analyses were performed using GraphPad Prism software version 7.0 (GraphPad Inc., La Jolla, CA, USA) and IBM SPSS Statistics 19 (IBM Corp., Armonk, NY, USA).

## Results

**Fig. 6:**
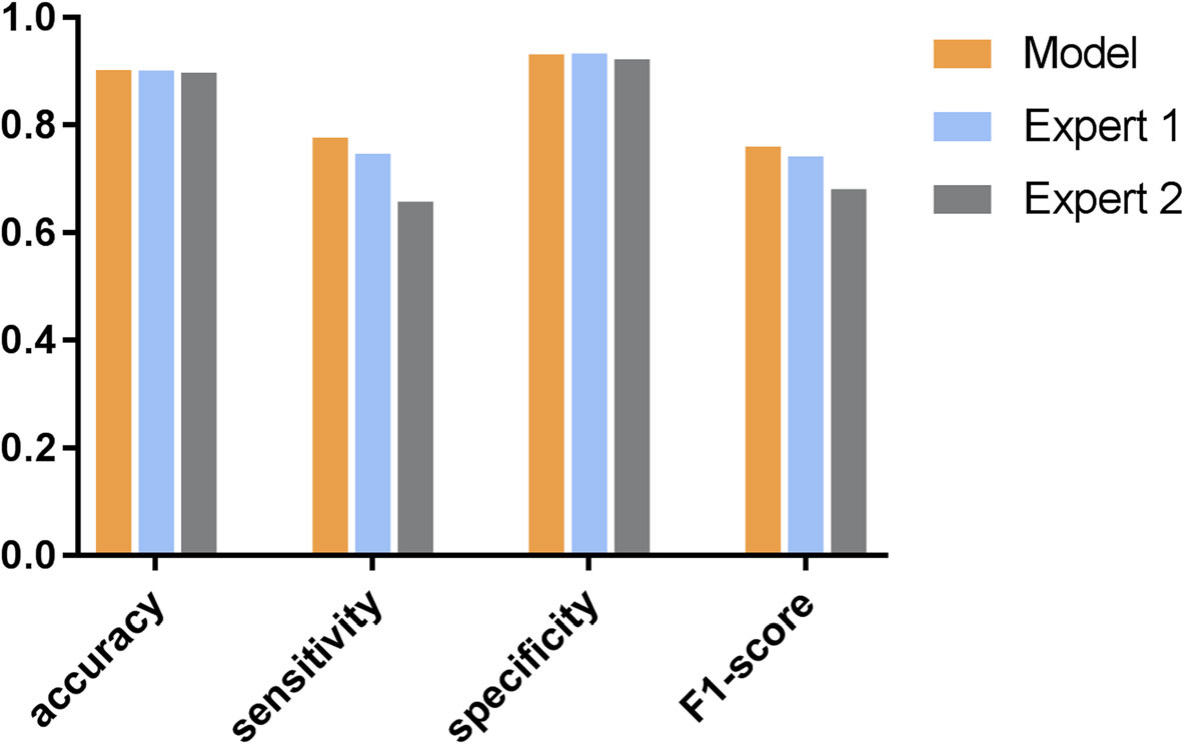
Performances of the proposed system and the two human experts for the four-degree classification of the ROP severity. The proposed system demonstrated 0.903 accuracy, 0.778 sensitivity, 0.932 specificity and 0.761 F1-score for the four-degree classification task; Expert 1 achieved 0.902 accuracy, 0.748 sensitivity, 0.934 specificity and 0.743 F1-score, while expert 2 achieved 0.898 accuracy, 0.659 sensitivity, 0.923 specificity and 0.682 F1-score. Abbreviations: ROP, retinopathy of prematurity

Our intelligent system was evaluated regarding its ability to discriminate the four-degree classification of ROP from fundus images; The results showed that the system can achieve an accuracy of 0.903, a sensitivity of 0.778 with a specificity of 0.932 and a F1-score of 0.761 for grading the ROP cases as “normal,” “mild,” “semi-urgent,” and “urgent” (Fig. 6).

We further compared the performance of the system with the results obtained by two experienced retinal experts. Expert 1 achieved an accuracy of 0.902, a sensitivity of 0.748, a specificity of 0.934 and a F1-score of 0.743; expert 2 achieved an accuracy of 0.898, a sensitivity of 0.659, a specificity of 0.923 and a F1-score of 0.682 (Fig. 6). Table 2 shows the specific accuracies for each category obtained by the proposed system and the two retinal experts. The results showed that the system could correctly discriminate the four-degree classification with accuracies of 0.883, 0.900, 0.957, and 0.870, respectively.

**Table 2:** The specific accuracies for each category by the intelligent system and the two retinal experts

Category	System	Expert 1	Expert 2
Normal	0.870	0.929	0.914
Mild	0.883	0.874	0.869
Semi-urgent	0.900	0.882	0.889
Urgent	0.957	0.923	0.920

**Fig. 7:**
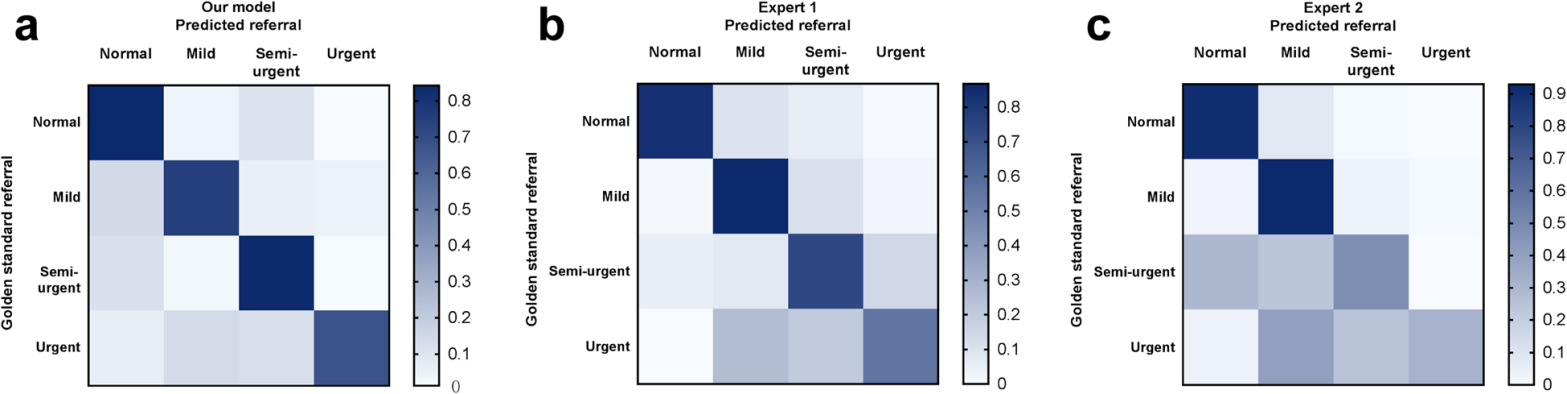
Three confusion matrixes for the intelligent system and the two best retina experts’ predictions in the four-degree classification task. (**a**) Confusion matrix of the proposed system; (**b**) Confusion matrix of expert 1; (**c**) Confusion matrix of expert 2

Three confusion matrixes shown in Fig. 7 reveal the specific assignments of different predictions for each image. The rows provide the samples’ true labels, while the columns present the predicted labels. Each diagonal element of the heatmap represents the percentage of images correctly classified in the corresponding category. Non-diagonal elements show the percentages of misclassified images and how they were misclassified. Misclassification cases and types were significantly fewer with the intelligent system than for human experts.

The accuracies of our system to identify the stage of ROP and the presence of plus disease were 0.957 and 0.896. Besides, it achieved an average F1-score of 0.78 in each category. Table 3 shows the specific accuracies, sensitivities and specificities of each category for the proposed system. The accuracies for discriminating stage I to stage V were 0.876, 0.942, 0.968, 0.998 and 0.999, respectively.

**Table 3:** The accuracies, sensitivities, specificities and F1-scores for each category by the proposed system

Definition	Clinical features	Accuracy	Sensitivity	Specificity	F1-score
Stage I	Demarcation line	0.876	0.765	0.883	0.81
Stage II	Ridge	0.942	0.550	0.973	0.52
Stage III	Ridge with extra retinal fibrovascular	0.968	0.473	0.975	0.51
Stage IV	Subtotal retinal detachment	0.998	0.867	0.998	0.93
Stage V	Total retinal detachment	0.999	0.800	0.999	0.89
Plus disease	Dilation and tortuosity of retinal vessels	0.896	0.713	0.907	0.78
Optic disc		0.954	0.945	0.917	0.96
Fovea		0.781	0.744	0.840	0.72
Laser scars		0.974	0.908	0.988	0.89

**Fig. 8:**
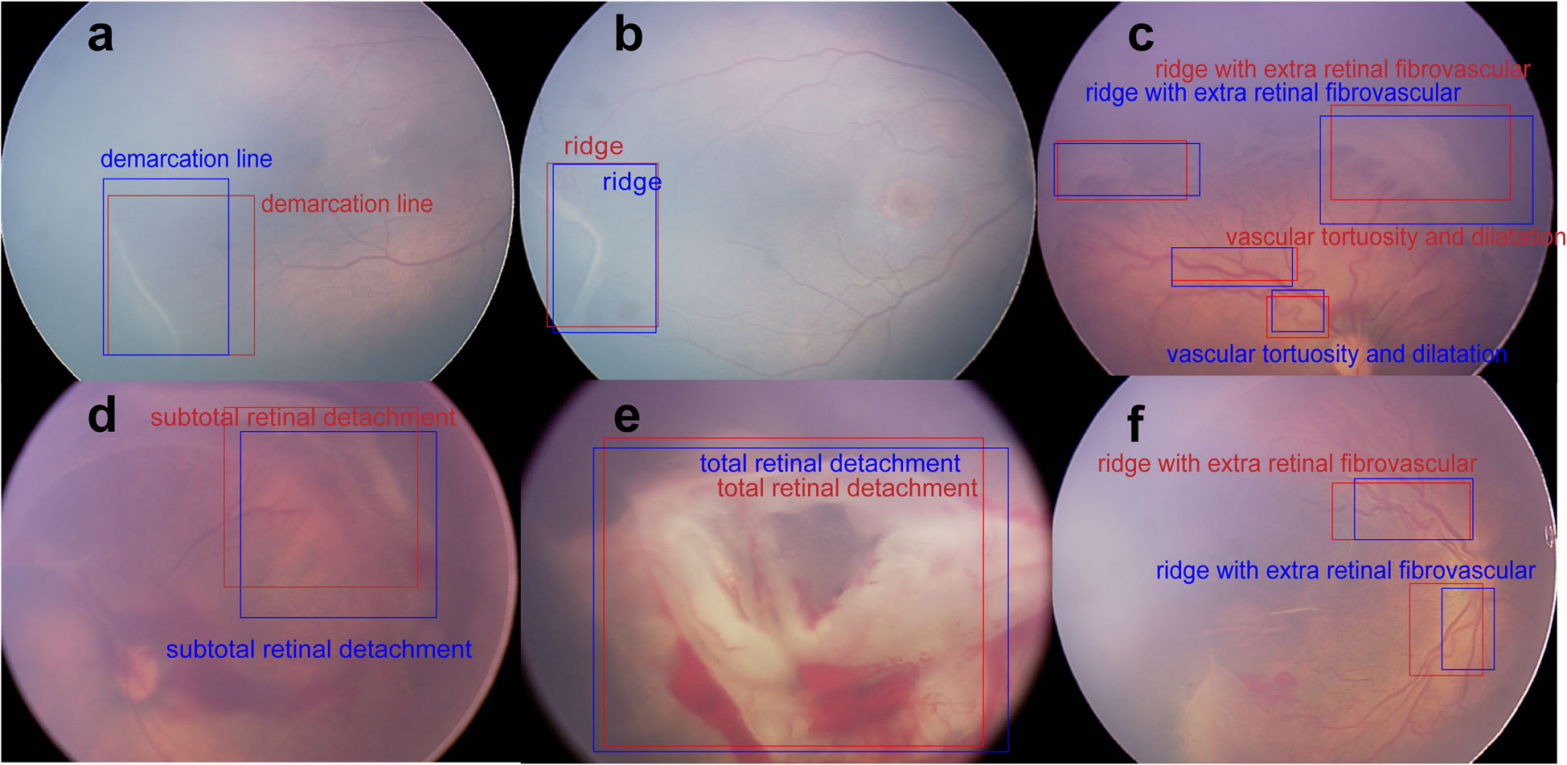
Representative images of system-predicted lesion locations compared with those predicted by experts. Box outlines in (**a**-**f**) indicate lesion sites. The boxes in red are the lesion locations annotated by retinal experts (gold standard annotations), and the boxes in blue are the lesion locations predicted by the Faster-RCNN. Abbreviation: Faster-RCNN, faster region-based convolutional neural network

Performance was also measured by evaluating whether the proposed outline boxes overlapped sufficiently with outline boxes that were provided as the gold standard. In the test phase, the re-trained model used test images as input then output the predicted category label and the outline box for each corresponding target object (Fig. 8).

## Discussion

We developed a new automated feature-learning approach for ROP detection using DL methods. This provides a robust solution for ROP detection within a large-scale annotated dataset, and the results showed high efficacy of the proposed model in providing objective and efficient ROP diagnosis without reliance on ophthalmologists for manual examination and grading of images. In addition to image classification, the system could accurately identify the stage of ROP and presence of plus disease, and could visualize abnormal regions, which are important for the clinical diagnosis of ROP.

By employing a transfer learning algorithm, the proposed system showed good performance for this application without the requirement for a highly specialized DL machine nor a novel database of millions of images. Key improvements are as follows: (1) a new dataset was constructed that is large and annotated with a new labeling scheme combining more clinical features of ROP, aiding in reduction of the individual effect and avoiding over-fitting of the algorithms to some specific feature; (2) two mainstream CNN models were applied as our classification and identification algorithms (101-layer ResNet and Faster-RCNN, respectively), which appeared to perform screening functions with proficiency comparable to or better than that of ROP experts; (3) the performance was optimized with a 10-fold cross-validation method that can increase the generalizability of the system.

Clinical studies have shown that zone I, any stage ROP with plus disease or zone I, stage III retinopathy without plus disease requires timely treatment to prevent blindness [29]. The most prominent advantage of our study is its attempt to identify the stage of ROP and presence of plus disease, along with disease severity; this functionality enables clinical review and verification of the automated diagnosis, rather than simply identifying the presence of ROP. Moreover, conventional deep neural networks (such as ResNet, AlexNet), provide only the image classification and associated labels without explicit definitions of features in clinical practice. Here, Faster-RCNN served as an object detection network that could recognize and classify object in an image and could position the object by using an outline box [27]; this enables ophthalmologists to inspect and visualize specific lesion regions. The algorithms developed in this study are advantageous in terms of the above properties when compared to other algorithms; the benefits also include consistent prediction and instantaneous reporting of results.

Previous studies of automated identification of ROP screening have shown encouraging results [6, 30, 31]. The majority of traditional methods for diagnosis of ROP are focused on the recognition of plus disease such as measuring the statistics of retinal vessels in the fundus [32]. For example, “ROPTool” and “i-ROP” systems were proposed to assist ophthalmologists in diagnosing plus disease [31, 33]. The ImageNet pre-trained GoogleNet was the first deep neural network to classify the presence of plus disease [34]. Although plus disease is an important clinical feature of ROP diagnosis by the International Classification of Retinopathy of Prematurity system defining treatment-requiring ROP, it is not sufficient to define ROP by itself only [21]. To the best of our knowledge, there has been minimal research focused on comparative analysis of image features that are most critical for diagnosis. In contrast to other studies, Wang J et al. developed a DL-based method and divided ROP into three grades with high sensitivity and specificity [35]. However, their system could only evaluate the severity of ROP; it could not identify finer details, such as the stage of ROP or presence of plus disease. Additionally, the numbers of the images in different datasets are insufficient to develop robust DL models that can deliver satisfactory performance [36]. An overview of previous studies using AI methods for ROP diagnosis are listed in Table 4**,** including a comparison of the dataset, diagnostic model and their applications in ROP.

**Table 4:** Prior ROP AI study performance comparison

Reference	Patients (N)	Cases	Images	Labels	Model	Specificity	Sensitivity	Accuracy
Wang et al. [35]	1273	3722	20,795	normal/minor	Id-Net; Gr-Net	99.32% (Id-Net);	96.62% (Id-Net); 88.46% (Gr-Net)	–
				ROP/severe ROP				
						92.31% (Gr-Net)		
Brown et al. [19]	898	1762	5511	normal/pre-plus	U-net (Inception-v1)	94% (plus disease)	93% (plus disease); 100% (pre-plus disease)	–
				disease/plus disease				
						94% (pre-plus disease)		
Worrall et al. [34]	35	347	1459	normal/plus disease	GoogleNet; Bayesian CNNs	0.983 (per image)	0.825 (per image)	–
							0.954 (per exam)	
						0.947 (per exam)		
Campbell et al. [37]	–	–	77	normal/pre-plus	i-ROP	–	–	95%
				disease/plus disease				
Hu et al. [38]	720	–	3017	normal/mild	Inception-v2; VGG-16; ResNet-50	–	–	0.970 (normal and ROP);
				ROP/severe ROP				
								0.840 (mild and severe)

*ROP* = retinopathy of prematurity; *GoogleNet* = google inception net; *CNN* = convolutional neural network; *VGG* = visual geometry group; *ResNet* = residual network

Some limitations of this study include: (1) limited number of ROP stage V fundus images in our dataset, which may have biased the performance of the model; (2) the fundus images in our study were collected from a single clinical site with consistent device settings and population characteristics, which might have reduced data diversity and affected the generalization ability of the algorithm; (3) our system struggled to differentiate between normal and very early cases of ROP in the dataset, such that it missed cases with subtle demarcation lines; (4) although we used a cross-validation method to maximize generalizability to other datasets, an important continuation to this study will be to achieve validation using completely separate images. Notably, premature infants who were diagnosed with avascular retina but no characteristics of ROP for the first screening time, needed to be followed up every 2–3 weeks until the retina was fully vascularized.

In future studies, larger datasets of severe ROP images are needed to validate and optimize our system in the clinical setting. Moreover, further testing and optimization of the sensitivity metric may be necessary to ensure a minimum false-negative rate. Additionally, multimodal clinical metadata should be included in the AI diagnosis of ROP, such as birth weight, patient history, gestational age, and other clinical data that may influence the risk of retinopathy. Datasets from multiple clinical centers and larger patient cohorts are needed in subsequent studies to further validate this intelligent system and enable it to serve as a practical intelligent tool for real-world clinical use.

## Conclusions

Overall, our DL-based system showed the potential for automated detection of ROP and differentiation of four-level classification fundus images with high accuracy and specificity. The performance of the system was equal to or better than that of retinal experts, suggesting that this system can be used to assist in clinical decisions; this is an initial step toward clinical translation of this method. Given the increasing burden of ROP on the healthcare system, the implementation of our algorithm is likely to be important in supporting decisions for patient management and primary care-based screening approaches for ROP in the general population.

## Data Availability

The dataset used and analyzed during the current study are available from the corresponding author on reasonable request.

## Abbreviations

ROP: Retinopathy of prematurity
Faster-RCNN: Faster region-based convolutional neural network
AI: Artificial intelligence
DL: Deep learning
CNN: Convolutional neural network
Conv: Convolutional layer
RPN: Region proposal network
ROI: Region of interest
AvgPool: Average pool
FC: Full connected layer
GoogleNet: Google inception net
VGG: Visual geometry group
ResNet: Residual network
